# Inflammation, Hypoxia, and Periodontal Breakdown: The Association Between Obstructive Sleep Apnea and Periodontitis; Results From a Systematic Review With Meta‐Analysis

**DOI:** 10.1002/cre2.70401

**Published:** 2026-07-19

**Authors:** Fariba Esperouz, Mauro Lorusso, Giuseppina Raffaella Giannini, Sara Ruffino, Elena D'Angelo, Michele Tepedino, Giuseppe Burlon, Francesca Sfondrini, Lucio Lo Russo, Domenico Ciavarella

**Affiliations:** ^1^ Department of Clinical and Experimental Medicine University of Foggia Foggia Italy; ^2^ Department of Biotechnological and Applied Clinical Sciences, Dental School of L'Aquila University of L'Aquila L'Aquila Italy; ^3^ Orthodontics and Pediatric Dentistry Unit, Section of Dentistry, Department of Clinical, Surgical, Diagnostic and Pediatric Sciences University of Pavia Pavia Italy

**Keywords:** obstructive sleep apnea, periodontal disease, periodontitis, sleep‐disordered breathing

## Abstract

**Objective:**

This systematic review and meta‐analysis aimed to evaluate the association between obstructive sleep apnea and periodontitis and to quantify the magnitude of this relationship based on the available evidence.

**Methods:**

A systematic literature search was conducted in PubMed, Scopus, and Web of Science up to November 2025, following PRISMA 2020 guidelines. Observational studies investigating the association between OSA and periodontitis in human adults were included. Study quality was assessed using the ROBINS‐I tool. A random‐effects meta‐analysis was performed to pool odds ratios (ORs) with 95% confidence intervals (CIs). Additionally, Spearman correlation coefficients were synthesized when available.

**Results:**

Twenty‐three studies were included in the qualitative synthesis, and 18 studies were eligible for quantitative analysis. The pooled meta‐analysis demonstrated that OSA was significantly associated with an increased risk of periodontitis (OR = 2.22; 95% CI: 1.78–2.77), with moderate heterogeneity (*I* ^2^ = 57.4%). Furthermore, a random‐effects meta‐analysis of correlation coefficients revealed a moderate positive association between OSA and periodontal disease severity (*r* = 0.39; 95% CI: 0.17–0.58). These findings were consistent across different populations and diagnostic approaches, particularly in studies using polysomnography for OSA assessment.

**Conclusions:**

The present meta‐analysis provides evidence of a significant association between obstructive sleep apnea and periodontitis, with patients affected by OSA showing more than twice the risk of developing periodontal disease. Although causality cannot be established due to the observational nature of the included studies, shared inflammatory and microbiological pathways support a biologically plausible link. An interdisciplinary approach involving dental and sleep medicine professionals may be beneficial for early diagnosis and comprehensive management of both conditions.

## 1 Introduction

Periodontitis is a chronic inflammatory disease involving the supporting tissues of the teeth, including the gums, periodontal ligament, root cementum, and alveolar bone and is caused by a dysregulated immune response to a complex bacterial biofilm. The interaction between the oral microbiota and the immune system leads to progressive tissue destruction, with the formation of periodontal pockets, loss of clinical attachment, and bone resorption (Mukherjee and Galgali 2021). Systemic and behavioral risk factors, such as smoking, diabetes, obesity, genetic predisposition and oxidative stress, influence the progression of the disease. Periodontitis is also associated with systemic consequences, including cardiovascular disease and metabolic disorders (Latorre et al. 2018). Recent studies have suggested that the composition of the oral microbiota may play a key role in the severity of periodontitis (Chen et al. 2023; Téllez‐Corral et al. 2022; Téllez Corral et al. 2023). The oral microbiota of a periodontal patient is often dominated by pathogenic species such as *Porphyromonas gingivalis, Tannerella forsythia,* and *Treponema denticola*. In particular, it has been shown that emerging microorganisms, such as species of the genus *Prevotella* and *Candida albicans*, are more prevalent in patients with advanced periodontal disease (Kozak and Pawlik 2023). Other studies show that periodontitis is associated with persistent systemic inflammation, characterized by elevated levels of IL‐1β, IL‐6, TNF‐α, and C‐reactive protein, as well as changes in saliva characteristics, such as reduced flow, altered pH, and increased inflammatory markers (Gamsiz‐Isik et al. 2017; Tranfić Duplančić et al. 2022).

Obstructive sleep apnoea (OSA) syndrome is characterized by recurrent episodes of partial or complete obstruction of the upper airways during sleep, resulting in intermittent hypoxia, hypercapnia, micro‐awakenings, and sleep fragmentation. These effects lead to activation of the sympathetic nervous system and a significant systemic inflammatory burden (Seo et al. 2013; Ciavarella and Spinoso 2025). Predisposing factors include obesity, increased cervical fat, muscle hypotonia, unfavorable craniofacial conformation, and anatomical airway restrictions. (Ashraf et al. 2022; Ciavarella et al. 2023) The intermittent hypoxia characteristic of OSA induces oxidative stress, endothelial dysfunction, and an increase in pro‐inflammatory cytokines, explaining the association with cardiovascular disease, insulin resistance, and neurocognitive disorders (Sales‐Peres et al. 2016; Ciavarella et al. 2025). Recent studies have highlighted local alterations in the oral cavity of OSA patients, including xerostomia, pH variations, and changes in the oral microbiota (Téllez‐Corral et al. 2022; Tranfić Duplančić et al. 2022), suggesting a possible impact of oral breathing and intermittent hypoxia on periodontal health.

The hypothesis of a link between periodontitis and OSA is supported by the convergence of several shared pathophysiological mechanisms. Both conditions are characterized by chronic inflammation, oxidative stress, and alterations in the microbiota, elements that can reinforce each other. The intermittent hypoxia typical of OSA amplifies the production of reactive oxygen species and promotes a systemic increase in pro‐inflammatory cytokines, including IL‐6, TNF‐α, and IL‐1β. This systemic inflammatory state may contribute to exacerbating periodontal inflammation, already sustained by a dysregulated immune response and the predominance of pathogenic bacteria in the gingival crevice. At the same time, periodontitis is a continuous source of inflammatory mediators and transient bacteraemia, which can contribute to worsening endothelial dysfunction and systemic inflammatory response in patients with OSA (Seo et al. 2013). At the local level, conditions frequently found in individuals with OSA—mouth breathing, xerostomia, and changes in salivary pH—promote an oral environment that is more susceptible to the development of periodontopathogen anaerobic species, leading to increased oral dryness and changes in salivary properties in patients with obstructive apneas (Tranfić Duplančić et al. 2022). Similarly, it has been shown that individuals with OSA have a higher prevalence of P. melaninogenica and Candida albicans, indicative of oral dysbiosis that could promote the progression of periodontal disease (Téllez‐Corral et al. 2022). Intermittent hypoxia itself can alter the physiology of periodontal tissues, promoting changes in cellular metabolism, reduction of antioxidant defences, and amplification of the local inflammatory response (Sales‐Peres et al. 2016). Similarly, periodontitis—through increased circulating levels of inflammatory cytokines—may contribute to worsening the systemic immune‐inflammatory activation that characterizes patients with OSA. This is supported by significantly higher levels of salivary IL‐6 in subjects with OSAS compared to controls, along with a correlation between periodontal parameters and OSA severity. These data suggest the existence of a vicious circle in which OSA and periodontitis may influence each other: on the one hand, OSA may predispose or aggravate periodontal disease through environmental and immuno‐inflammatory alterations; on the other hand, periodontitis—through its systemic inflammatory component—could contribute to amplifying the endothelial dysfunction and oxidative stress typical of OSA (Nizam et al. 2016).

The aim of this meta‐analysis is to understand whether, in light of the results of the currently available literature, there is a correlation between periodontal disease and OSA and, if so, to what extent.

## 2 Materials and Methods

This systematic review and meta‐analysis were conducted in accordance with the *Preferred Reporting Items for Systematic Reviews and Meta‐Analyses (PRISMA 2020)* guidelines (Page et al. 2021). The protocol for this review was prospectively registered in the International Prospective Register of Systematic Reviews (PROSPERO) under the registration number CRD420251241487.

### 2.1 Search Strategy

A comprehensive literature search was conducted in PubMed, Scopus, and Web of Science (WOS) to identify studies investigating the association between obstructive sleep apnea (OSA) and periodontitis. The search included all available records up to November 2025, with no restrictions on publication year. Only studies published in English were included during the screening process. The PubMed search strategy combined MeSH terms and free‐text words using Boolean operators as follows: (“Sleep Apnea, Obstructive” [MeSH Terms] OR “obstructive sleep apnea” [Text Word] OR OSA [Text Word] OR OSAS [Text Word]) AND (“Periodontitis” [MeSH Terms] OR periodontitis [Text Word] OR “periodontal disease” [Text Word] OR “periodontal diseases” [MeSH Terms]). Equivalent search strategies were adapted for Scopus and WOS. Additionally, the reference lists of relevant systematic reviews were manually screened to identify any further eligible studies.

### 2.2 Eligibility Criteria

All retrieved studies were imported into a reference management software, and duplicates were removed. The titles and abstracts of all unique records were screened to determine eligibility according to predefined inclusion and exclusion criteria.

#### 2.2.1 Inclusion Criteria

Studies were included if they met the following criteria: (1) Studies were eligible for inclusion only if they involved adult participants aged 18 years or older; (2) published in English; (3) no limits were imposed on the year of publication; (4) employed an observational study design, including cross‐sectional, case–control, cohort, or longitudinal approaches; (5) investigated the association between obstructive sleep apnea (OSA) and periodontitis in human populations; (6) reported quantitative data or effect estimates (e.g., odds ratios, risk ratios) suitable for inclusion in a meta‐analysis; and (7) used validated diagnostic methods for both OSA, including polysomnography, home sleep/cardiorespiratory monitoring with Apnea–Hypopnea Index (AHI) assessment, or validated screening questionnaires such as STOP‐BANG and Berlin questionnaires.

#### 2.2.2 Exclusion Criteria

Studies were excluded if they met any of the following criteria: (1) they were not published in English; (2) they were reviews, meta‐analyses, case reports, conference abstracts, or editorials; (3) they were animal or in vitro studies; (4) they did not provide extractable quantitative data suitable for meta‐analysis, or reported only continuous periodontal measures (e.g., probing depth, clinical attachment loss, bleeding on probing) without presenting prevalence data or effect estimates, such as odds ratios or risk ratios; (5) they included duplicated data sets that were already represented in more recent or more comprehensive publications; or (6) they did not directly assess the association between OSA and periodontitis.

### 2.3 Focused PICO Question and Outcome Measures

The population (P) consisted of human participants in whom both OSA and periodontal status were assessed. The exposure (I) of interest was the presence or severity of OSA, determined using validated diagnostic approaches such as polysomnography or standardized questionnaires. The comparison (C) group included participants without OSA or with subclinical levels of apnea, according to study‐specific thresholds. Outcomes (O) encompassed the occurrence or prevalence of periodontitis, measures of periodontal health (including probing pocket depth, clinical attachment loss, bleeding on probing, and alveolar bone loss), and statistical associations between OSA and periodontal parameters, expressed as odds ratios, risk ratios, or correlation coefficients (Spearman or Pearson).

To be eligible for inclusion in the quantitative synthesis, studies were required to report either prevalence data, effect estimates (ORs or RRs), continuous periodontal measurements with accompanying measures of variability (mean ± SD), or correlation coefficients suitable for meta‐analytic pooling.

### 2.4 Studies Screening and Inclusion

Two authors (G.G. and S.R.) independently screened all retrieved citations by evaluating titles and abstracts against the predefined inclusion and exclusion criteria. After this initial screening, potentially eligible studies were selected for full‐text review to assess their suitability for inclusion in the qualitative and quantitative synthesis. In cases of disagreement or uncertainty, a third independent reviewer (F.E.) was consulted to reach a final consensus.

### 2.5 Data Extraction

Data extraction was performed independently by two reviewers using a standardized data collection form. Extracted variables included: first author and year of publication, country, study design, sample size, participants’ age range and mean age, methods used for the diagnosis of OSA and for periodontitis, and quantitative outcomes (Table 1). Discrepancies between reviewers were resolved through discussion or consultation with a third reviewer. When relevant data were missing or incomplete, attempts were made to contact the corresponding authors of the original studies to obtain additional information.

**Table 1:** Summary description of the included studies.

ID	Year	Country	Study design	Sample size		Age range	Mean age (years)	OSA variables	Periodontal variables	Results
				Case	Control					
Ahmad	2013	North Carolina, USA	Case‐Control study	50	104	NR	61	STOP questionnaire	PPD and CAL	Moderate or severe periodontitis was significantly associated with a higher risk for OSA
Keller	2013	Taiwan	Case‐control study	7321	21,963	18–70	47.6 ± 15.4	PSG	Periodontal examination, PD, Mobility, and ABL	OSA was significantly associated with a history of chronic periodontitis, with a higher prevalence of prior CP observed in OSA patients compared to controls, independent of comorbidities and socioeconomic factors.
Seo	2013	Korea	Cross sectional study	320	267	47–77	55.85 ± 6.63	PSG and AHI	CAL and PPD	OSA was positively associated with periodontitis and its severity, with stronger associations observed in participants aged 55 years and above.
Loke	2014	Texas	Cross‐sectional study	74	26	28–79	52,6	PSG and AHI	CAL and PPD	OSA severity was not significantly associated with moderate/severe periodontitis or most periodontal parameters, except for a higher percentage of sites with plaque.
Sanders	2015	USA	Cross‐sectional study	12,469		18–74		Home sleep test (ARES)	PD, CAL and GENGIVAL RECESSION	The study found that sleep‐disordered breathing was independently associated with severe chronic periodontitis: even subclinical breathing disturbances corresponded to 40% higher adjusted odds, mild SDB to 60% higher odds, and moderate to severe SDB to 50% higher odds of severe periodontitis compared with non‐apneic subjects.
Al‐Habashneh	2016	Jordan	Cross‐sectional study	296		30–60	40 ± 8.5	Berlin questionnaire	PPD and CAL	Periodontitis was about twice as prevalent among individuals at high risk for OSA, and habitual snoring showed an independent association with periodontal disease.
Sales‐Peres	2016	Brazil	Cross‐sectional study	108		30–60	40,1	Berlin questionnaire and ESS	CAL, BOP, and PD	In Class III obese patients, periodontal disease was not associated with OSAS risk, which was instead linked to increased neck circumference and predicted neck circumference.
Nizam	2016	Turkey	Case‐control study	39	13	21–64	46.60	PSG and AHI	PD, CAL, PPD, BOP, OHI‐S, GI, and GR	OSAS was associated with altered subgingival microbiota, increased salivary IL‐6, and greater periodontal disease severity, supporting a potential link between OSAS and periodontitis.
Gamsiz‐Isik	2017	Turkey	Case‐control study	83	80	30–68	45.58 ± 9.12	PSG and AHI	PI, GI, BOP, PD, and CAL	Patients with OSA exhibited higher prevalence and severity of periodontitis, elevated GCF IL‐1β, and increased serum hs‐CRP, highlighting a potential inflammatory link between OSA and periodontal disease.
Kale	2018	India	Cross‐sectional study	130	130	21–72	43.67 ± 11.89	STOP and BANG questionnaire	CPI and CAL	Periodontitis did not differ significantly between high‐ and no‐risk OSA groups, suggesting no clear association in this cohort.
Latorre	2018	Colombia	Cross‐sectional study	199		30–85	49.9	PSG	PD, CAL,and PPD	Periodontitis was associated with mild OSA in women with hypertension or hypertensive cardiomyopathy, and with severe OSA in men with similar comorbidities, highlighting sex‐ and comorbidity‐specific links between periodontal disease and OSA.
Chen	2021	China	Cross‐sectional study	27	27	24–35	28.5 ± 3.1	PSG	PD and CAL	OSA was associated with reduced salivary microbial richness and increased abundance of Prevotella, suggesting a link between altered oral microbiota and higher periodontitis prevalence in OSA patients.
Pico‐Orozco	2021	Spain	Case control study	60	54	27–71	52.9 ± 10.2	PSG, RP, and AHI	PD, CAL, BOP, PI, and CI	Periodontitis prevalence and severity were higher in sleep apnea‐hypopnea syndrome (SAHS) patients, particularly in those with obesity, highlighting BMI as a key factor and supporting periodontal screening in obese SAHS patients.
Mukherjee	2021	India	Cross‐sectional study	109	141	18–80	42.5	STOP‐BANG questionnaire	CAL and PPD	A moderate positive association was observed between periodontitis and OSA, with higher PPD and CAL scores in subjects at increased OSA risk.
Ashraf	2022	India	Case‐control study	60	60	18–77	49.08 ± 13.13	STOP‐Bang, PSG, and ESS	CPI‐modified and CAL	Periodontal disease, particularly mild to moderate periodontitis, was more prevalent in OSA patients, supporting a possible bidirectional inflammatory association between OSA and periodontitis.
Tellez‐Corral	2022	Colombia	Cross sectional study	93		30–72	46.9	PSG and AHI	PD, CAL, BOP, and PI	Prevotella melaninogenica and Candida albicans altered the oral microbiota in patients with periodontitis and OSA, potentially increasing periodontal disease severity and contributing to the high prevalence of stage III periodontitis in severe OSA.
Verhelst	2022	Netherlands	Cross sectional study	70	77	≥ 40	53.9	Philips Questionnaire	CAL and PPD	Periodontitis patients were more frequently classified as high risk for OSA, suggesting that targeted screening in this population may aid early identification of OSA risk.
Tranfić Duplančić	2022	Croatia	Cross‐sectional study	188	17	31–68	57.5	PSG and PG	REC, PPD, BOP, CAL, PISA, and PI	OSA severity was associated with increased plaque and higher CAL, while salivary flow and composition remained largely unchanged, suggesting that OSA may influence periodontal inflammation through multifactorial interactions rather than direct changes in saliva.
Stazic	2022	Croatia	Cross‐sectional study	194		NR	NR	PSG or PG, AHI, ODI, and ESS	CAL, PPD, GE, FMBS, FMPS	OSA was associated with more severe periodontitis, with additional risk factors including older age, smoking, infrequent dental visits, and poor oral hygiene, supporting the need for periodontal screening in patients with severe OSA.
Chen	2023	China	Cross‐sectional study	51	42	24–35	28.8 ± 3.0	PSG, AHI, and ODI	PD‐CAL and BOP	OSA was significantly associated with periodontitis, with low oxygen saturation emerging as a potential predictor, suggesting a link between hypoxia and periodontal disease severity.
Ytzhaik	2023	Israel	Cross‐sectional study	318	132,211	18–50	NR	PSG and AHI	PD, CAL, and BOP	OSA was positively associated with periodontal disease, alongside obesity, male sex, smoking, and age, supporting the integration of dental evaluation in OSA risk management and highlighting the interplay between oral and systemic health.
Tellez ‐Corral	2023	Colombia	Cross‐sectional study	75		> 30	46.8	PSG	PD, CAL, BOP, and PI	Cryptic oral microorganisms are associated with periodontitis and OSA, potentially promoting dysbiotic oral microbiota and contributing to the shared pathophysiology of these conditions.
Florian‐Tirado	2024	Peru	Cross‐sectional study	118		18–70	45.18 ± 14.57	STOP‐BANG questionnaire	CAL and PPD	Periodontitis was significantly associated with OSA risk in Peruvian adults, with additional links observed with diabetes and asthma, but not with age or sex.

Abbreviations: ABL, Alveolar Bone Loss; BOP, Bleeding on Probing; AHI, Apnea‐Hypopnea Index; CAL, Clinical Attachment Level; CI, Calculus Index; CPI and CPI‐modified, Community Periodontal Index; ESS, Epworth Sleepiness Scale; FMBS, Full Mouth Bleeding Score; FMPS, Full Mouth Plaque Score; GI, Gingival Index; GR, Gingival Recession; ODI, Oxygen Desaturation Index; OHI‐S, Oral Hygiene Index – Simplified; PD, Periodontal Disease; PG, portable polygraphy for sleep assessment; PSG, polysomnography; PPD, Probing Pocket Depth; PI, Plaque Index; PISA, Periodontal Inflamed Surface Area; RP, respiratory polygraphy; STOP, Snoring, Tiredness, Observed apnea, high blood Pressure questionnaire; STOP‐Bang, STOP tool by adding BMI, age, neck circumference, and gender.

### 2.6 Assessment of Risk of Bias

The assessment of study quality and risk of bias was conducted using the ROBINS‐I tool (Risk of Bias in Non‐randomized Studies of Interventions), which evaluates the domains of selection, comparability, and outcomes for each included study. Studies rated as having critical or serious concerns were considered at high risk of bias (Sterneet et al. 2016). Two reviewers (G.G. and S.R.) independently carried out the assessment, and any disagreements were resolved through discussion with a third reviewer (FE).

### 2.7 Statistical Analysis

Studies were considered eligible for quantitative synthesis when they provided sufficient and extractable numerical data regarding the association between OSA and periodontitis, including effect estimates or raw data suitable for meta‐analysis. Studies lacking adequate statistical information or reporting incompatible outcomes were included only in the qualitative synthesis. Because of the limited number of available studies and the heterogeneity of reported outcomes, studies using objective OSA diagnostic methods (e.g., polysomnography or AHI‐based assessment) and studies using validated questionnaire‐based assessments were pooled together in the quantitative analysis

The statistical analysis was conducted by combining the effects of the included studies using a meta‐analysis based on odds ratios (OR) with 95% confidence intervals (95% CI), as the outcome considered was dichotomous. The ORs were obtained from the respective articles (Ahmad et al. 2013; Al Habashneh et al. 2016; Sanders et al. 2015; Ashraf et al. 2022; Chen et al. 2021; Chen et al. 2023; Gamsiz‐Isik et al. 2017; Kale et al. 2018; Keller et al. 2013; Latorre et al. 2018; Loke et al. 2015; Pico‐Orozco et al. 2021; Sales‐Peres et al. 2016; Seo et al. 2013; Téllez Corral et al. 2023; Téllez‐Corral et al. 2022; Verhelst et al. 2022; Ytzhaik et al. 2023; McGuinness and Higgins 2021). For each study, the OR was calculated using the Inverse Variance (IV) method, which assigns greater weight to studies with less variance. Since methodological and clinical variability between studies was expected, the synthesis of effects was performed using a random‐effects model. This model assumes that studies do not share a single true common effect, but that each estimate slightly different effects around a distribution of values. Heterogeneity between studies was assessed using three complementary metrics: (1) Cochran's *Q* statistic (*χ* ^2^), which tests whether the observed variability between the effects studied is greater than that expected for sampling variability alone; (2) the estimate of variance between studies (*τ* ^2^), used within the random effects model to measure how much variability exists between studies; (3) the *I* ^2^ index, which quantifies the total variability attributable to real differences between studies, and not to chance. All studies were visually represented using forest plots, in which each effect is accompanied by the relative 95% CI and the weight attributed by the statistical model. The overall effect was estimated using the weighted average of the individual effects, calculated according to the random effects model. The analytical process was carried out using software dedicated to meta‐analytical analysis (R, with the meta and metafor packages), which allows the automatic calculation of ORs, weights, measures of heterogeneity, and the estimate of the overall effect.

The statistical analysis also incorporated the synthesis of Spearman correlation coefficients (*r*) reported directly by several of the included studies (Mukherjee and Galgali 2021; Tranfić Duplančić et al. 2022; Nizam et al. 2016; Florian‐Tirado WL et al. 2024). For the study by Stazić et al. (2022), which reported Pearson correlation coefficients, the values were converted to their Spearman equivalents using the standard transformation:

(1) $$r_{s}\approx \frac{6}{\pi }\;\arcsin\left(\frac{r_{p}}{2}\right).$$

This allowed all correlation measures to be expressed on a consistent scale (Gilpin 1993). Since the coefficient *r* is not normally distributed, Fisher's transformation (*z*) was applied to combine the effects, which stabilizes the variance and allows the use of parametric methods in meta‐analysis. The overall estimate was obtained using the inverse variance (IV) method. Given the possible methodological variability between studies (different populations, measurement tools, and experimental conditions), a random‐effects model was used to combine the correlations. Heterogeneity was assessed using three complementary measures: (1) Cochran's *Q* statistic (*χ* ^2^); (2) *τ* ^2^; and (3) *I* ^2^. Each study included was represented graphically using a forest plot, which shows the relative 95% confidence intervals and the weight assigned by the model. The overall effect was obtained by combining the transformed estimates; subsequently, the value obtained was converted back using back‐transformation to return the interpretable correlation *r*. Again, all analyses were performed using meta‐analysis software, employing specific functions for the quantitative synthesis of correlations.

## 3 Results

### 3.1 Study Selection

**Figure 1:**
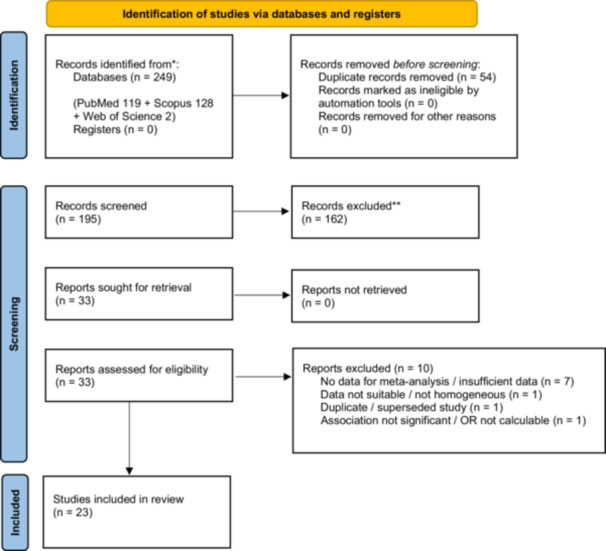
PRISMA flowchart (McGuinness and Higgins 2021).

The database search identified 249 records (PubMed: 119; Scopus: 128; WOS: 2). In addition, the reference lists of three prior systematic reviews (Portelli et al. 2024; khodadadi et al. 2022; Zhang et al. 2022) were manually screened, resulting in the identification of five further eligible studies: Chen et al. (2021), Pico‐Orozco et al. (2021), Ytzhaik et al. (2023), Kale et al. (2018), and Sanders et al. (2015). After removing 54 duplicates, 195 unique records remained for title and abstract screening. Of these, 162 were excluded as they clearly did not meet the inclusion criteria. The full texts of the remaining 33 articles were then assessed in detail, leading to the exclusion of 10 studies due to insufficient or non‐extractable data, heterogeneous outcome measures, irrelevance to the research question, or duplication of previously reported cohorts. Following this process, a total of 23 studies were included in the qualitative and quantitative synthesis, including the four studies identified from prior systematic reviews. The selection process is summarized in the PRISMA 2020 flow diagram (Figure 1).

### 3.2 Risk of Bias Assessment

**Figure 2:**
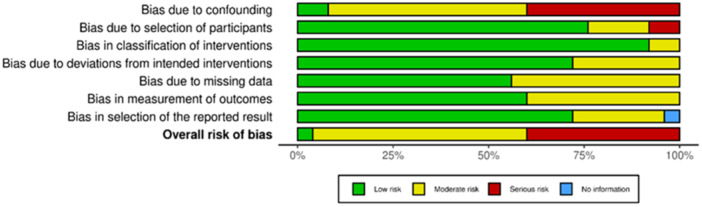
Risk of bias assessment of the 23 studies included in the review. The evaluation was conducted using the ROBINS‐I tool, and the summary plot was generated with the robvis package (Page et al. 2021).

Overall, the methodological quality of the included studies ranged from low to serious risk of bias (Figure 2). Most investigations were judged at moderate overall risk (Mukherjee and Galgali 2021; Chen et al. 2023; Téllez‐Corral et al. 2022; Téllez Corral et al. 2023; Gamsiz‐Isik et al. 2017; Seo et al. 2013; Ashraf et al. 2022; Sales‐Peres et al. 2016; Nizam et al. 2016; Ahmad et al. 2013; Keller et al. 2013; Pico‐Orozco et al. 2021; Ytzhaik et al. 2023; Sanders et al. 2015; Stazić et al. 2022). A smaller group of studies showed a low overall risk of bias (Tranfić Duplančić et al. 2022; Chen et al. 2021; Loke et al. 2015; Verhelst et al. 2022), while several were classified as having a serious overall risk (Latorre et al. 2018; Al Habashneh et al. 2016; Kale et al. 2018; Florian‐Tirado WL et al. 2024). Overall, the included studies exhibited a heterogeneous risk‐of‐bias profile, with ratings spanning from low to serious across both the total assessment and the individual ROBINS‐I domains.

### 3.3 Study Characteristics

The included studies (Table 1) were conducted across multiple countries, including the United States (Ahmad et al. 2013; Sanders et al. 2015), Jordan (Al Habashneh et al. 2016), India (Mukherjee and Galgali 2021; Ashraf et al. 2022; Kale et al. 2018), China (Chen et al. 2023; Chen et al. 2021), Turkey (Gamsiz‐Isik et al. 2017; Nizam et al. 2016), Taiwan (Keller et al. 2013), Colombia (Latorre et al. 2018; Téllez‐Corral et al. 2022; Téllez Corral et al. 2023), Spain (Pico‐Orozco et al. 2021), Israel (Ytzhaik et al. 2023), Brazil (Sales‐Peres et al. 2016), Korea (Seo et al. 2013), Peru (Florian‐Tirado WL et al. 2024), the Netherlands (Verhelst et al. 2022), and Croatia (Tranfić Duplančić et al. 2022; Stazić et al. 2022), published between 2013 and 2024. Sample sizes varied considerably, ranging from 52 to 132,354 participants, with age ranges spanning 18 to over 85 years. The majority of studies employed a cross‐sectional design (Mukherjee and Galgali 2021; Seo et al. 2013; Sales‐Peres et al. 2016; Al Habashneh et al. 2016; Kale et al. 2018; Chen et al. 2021; Ytzhaik et al. 2023; Loke et al. 2015; Sanders et al. 2015; Verhelst et al. 2022; Florian‐Tirado WL et al. 2024; Stazić et al. 2022), while a subset utilized a case‐control design (Gamsiz‐Isik et al. 2017; Ashraf et al. 2022; Nizam et al. 2016; Ahmad et al. 2013; Keller et al. 2013; Pico‐Orozco et al. 2021).

OSA assessment was performed using both subjective and objective methods, including questionnaires such as STOP, STOP‐Bang, Berlin, and Philips Questionnaires, as well as polysomnography (PSG), portable polygraphy (PG or RP), and indices including the Apnea‐Hypopnea Index (AHI), Oxygen Desaturation Index (ODI), and Epworth Sleepiness Scale (ESS).

The included studies employed different approaches to classify periodontal disease, largely reflecting the year of publication and the evolution of diagnostic criteria. Studies published before 2017 predominantly used classical clinical measures, such as Probing Pocket Depth (PPD), Clinical Attachment Level (CAL), Bleeding on Probing (BOP), Plaque Index (PI), Gingival Index (GI), Alveolar Bone Loss (ABL), and tooth mobility, or applied Page & Eke's criteria for epidemiological case definitions (Sales‐Peres et al. 2016; Nizam et al. 2016; Ahmad et al. 2013; Al Habashneh et al. 2016; Keller et al. 2013; Loke et al. 2015). In contrast, studies conducted after 2017 increasingly adopted the 2017 World Workshop on the Classification of Periodontal and Peri‐Implant Diseases and Conditions (Caton et al. 2018), which introduces a staging and grading system based on disease severity, complexity, and progression risk (Chen et al. 2021; Téllez‐Corral et al. 2023, 2022; Tranfić Duplančić et al. 2022; Ashraf et al. 2022; Chen et al. 2021; Florian‐Tirado et al. 2024). This distinction highlights the methodological shift in recent research toward a more standardized and comprehensive assessment of periodontitis, facilitating comparisons across studies and allowing more precise evaluation of its association with obstructive sleep apnea.

### 3.4 Meta‐Analysis

**Figure 3:**
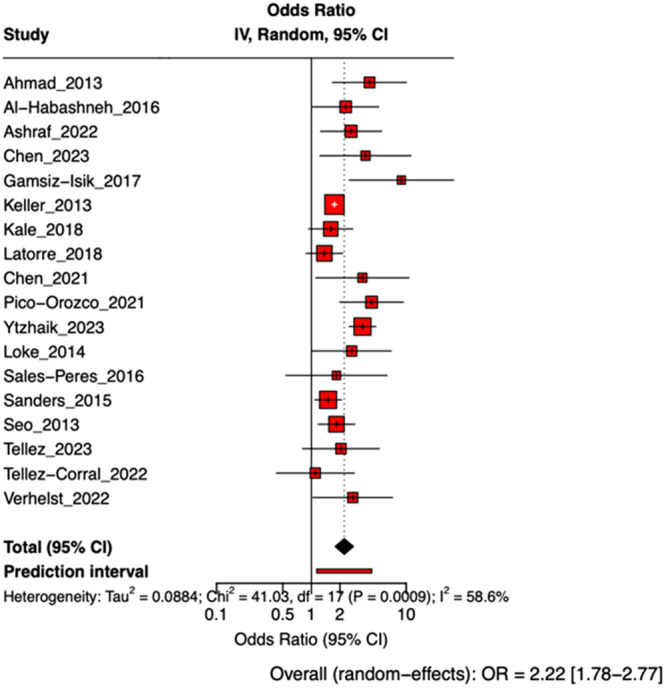
Forest plot of the random‐effects meta‐analysis reporting odds ratios (ORs) with 95% confidence intervals for the included studies.

A total of 18 studies were included in the quantitative synthesis. The pooled analysis showed that obstructive sleep apnea (OSA) was significantly associated with an increased likelihood of developing periodontitis, yielding a combined odds ratio of 2.22 (95% CI: 1.78–2.77). This finding, illustrated in the forest plot (Figure 3), indicates patients with OSA present a higher risk of periodontitis compared with non‐OSA individuals. There was moderate heterogeneity among the included studies (*I* ^2^ = 57.4%, *p* = 0.0013), suggesting variability in population characteristics and disease severity across studies. The prediction interval, which indicates the potential range of effects expected in future studies, also supported a positive association, highlighting the clinical relevance of these findings beyond statistical significance. Overall, these results reinforce the hypothesis that OSA may contribute to the development and progression of periodontal disease, likely mediated by systemic and local inflammatory pathways, oral breathing, and salivary changes that unfavorably affect periodontal tissues.

**Figure 4:**
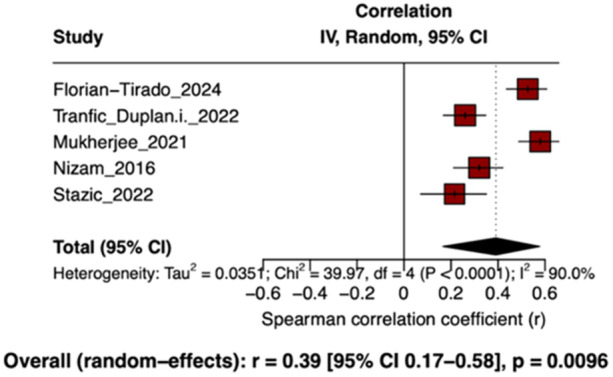
Forest plot depicting overall Spearman correlation coefficient estimation for meta‐analysis assessing the association between obstructive sleep apnea (OSA) and periodontitis. 95% CI: 95% Confidence Interval.

The random‐effects meta‐analysis of Spearman correlation coefficients, including five studies, showed a pooled correlation of *r* = 0.39 (95% CI: 0.17–0.58), consistent with a moderate positive association between the analyzed variables (Figure 4). Statistical heterogeneity was low to moderate (*I* ^2^ = 36.0%, *τ* ^2^ = 0.0152, *p* = 0.18), indicating that most variability among studies was likely due to sampling error rather than true differences in effect size. Overall, these results support a consistent moderate correlation across the included studies.

## 4 Discussion

The result obtained from this systematic review and meta‐analysis shows that there is a significant relationship between obstructive sleep apnea (OSA) and periodontitis. The odds ratio obtained was 2.22 (95% CI: 1.78–2.77), indicating that people with sleep apnea are more than twice as likely as people without sleep disorders to develop periodontitis. Moreover, the Spearman correlation coefficient result obtained with a value of *r* = 0.39 (95% CI: 0.15–0.58) shows a moderate and significant relationship between OSA and periodontitis, suggesting consistency in the association. Recent systematic reviews and meta‐analyses (Khodadadi et al. 2022; Portelli et al. 2024; Zhang et al. 2022) have further supported the association between obstructive sleep apnea and periodontitis, highlighting the potential role of chronic systemic inflammation, oxidative stress, cytokine dysregulation, oral microbiota alterations, and shared risk factors in the pathophysiological interaction between the two conditions. In particular, increased levels of IL‐1β, IL‐6, TNF‐α, hs‐CRP, and changes in oral microbial composition, including increased prevalence of *Prevotella* species, have been described in OSA patients with periodontal disease. The majority of cross‐sectional and case‐control studies included (Chen et al. 2023; Gamsiz‐Isik et al. 2017; Seo et al. 2013; Ashraf et al. 2022; Nizam et al. 2016; Ahmad et al. 2013; Al Habashneh et al. 2016; Pico‐Orozco et al. 2021; Ytzhaik et al. 2023; Sanders et al. 2015; Stazić et al. 2022) have shown an association with higher prevalence and severity of periodontitis and its clinical indices, like PD, CAL, and BOP, among patients with OSA. It has been noticed that there were relatively stronger associations among polysomnographic studies. It appears that an appropriate diagnosis of OSA can improve and make more specific the identification associated with periodontitis changes among patients with sleep disorders. Findings of current research are consistent with previous reports, as that of Gunaratnam et al. who were pioneering with a high prevalence periodontitis among patients with sleep disorders and thus documented an association with inflammation (Gunaratnam et al. 2009).

A clear biological link begins to appear among these studies. A number of trials have shown an augmentation of local and systemic inflammation as reflected either at the local level by levels of various factors within the gingival crevicular fluid or at the systemic level. To cite an example, Gamsiz‐Isik, among others, have shown an elevation of local and systemic levels of IL‐1β and hs‐CRP among patients with OSA (Gamsiz‐Isik et al. 2017), and Nizam and colleagues have shown an increase in salivary levels of IL‐6 (Nizam et al. 2016). Moreover, an interesting finding suggesting an association between nocturnal hypoxemia and periodontitis severity has been shown by Chen and colleagues, suggesting intermittent local hypoxia due to OSA as potentially contributing toward these factors (Chen et al. 2023).

Modifications in the oral microbiota could also act as an additional mediator. Findings from research works (Téllez‐Corral et al. 2022; Téllez Corral et al. 2023; Chen et al. 2021) have shown that there were lower microbial diversity, presence of cryptic species, and more periodontopathogens with an emphasis on Prevotella among patients with obstructive sleep apnea. Tranfiċ Duplaniċ et al have also seen an increase in dental plaques among patients with obstructive sleep apnea and have attributed it perhaps to mouth breathing, nocturnal xerostomia, and salivary dysfunction (Tranfić Duplančić et al. 2022).

Periodontitis is not limited to a local inflammatory process, but may also contribute to systemic immune activation (Martínez‐García and Hernández‐Lemus 2021). The ulcerated epithelium of periodontal pockets allows the translocation of bacterial products and inflammatory mediators into the bloodstream, promoting low‐grade systemic inflammation (Hajishengallis and Chavakis 2021). This mechanism may lead to increased circulating levels of pro‐inflammatory cytokines, including IL‐1β, IL‐6, TNF‐α, and C‐reactive protein, which are also involved in the pathophysiology of obstructive sleep apnea (Fan et al. 2025). In patients with OSA, intermittent hypoxia and sleep fragmentation further enhance oxidative stress and inflammatory pathway activation, potentially amplifying the systemic inflammatory burden generated by periodontal disease (Li et al. 2025). In addition to inflammatory mechanisms, recent evidence suggests that periodontitis and obstructive sleep apnea may also share common immunological and genetic susceptibility pathways (Incerti Parenti et al. 2025). Both conditions are characterized by dysregulated host immune responses, oxidative stress, and increased production of pro‐inflammatory cytokines. Genetic polymorphisms involving inflammatory mediators, particularly IL‐1, IL‐6, and TNF‐α, have been proposed as potential contributors to the susceptibility and progression of both diseases (Téllez Corral et al. 2023). Furthermore, alterations in immune regulation and host response may enhance tissue destruction and systemic inflammatory burden, supporting the hypothesis of a bidirectional biological relationship between periodontitis and OSA (Wu and Wang 2026).

The consistency of the association is also reinforced by the presence of shared systemic risk factors, including obesity, male sex, older age, smoking, and inadequate oral hygiene. Several studies (Latorre et al. 2018; Pico‐Orozco et al. 2021; Ytzhaik et al. 2023; Stazić et al. 2022) have emphasized the role of BMI, comorbid cardiovascular conditions, gender difference associations, and hygiene practices. Arango Jiménez et al. emphasize the mediating role of systemic inflammation, demonstrating that there are common risk factors for OSA and periodontitis via inflammation, and obesity and metabolic conditions aggravating inflammation and oxidative stress, that may accentuate periodontitis itself (Arango Jimenez et al. 2023). These common risk factors could explain, at least partially, these findings, but it seems that there is an association between periodontitis and OSA. Overall, despite some divergent findings, there would appear, from the included literature, an integrated and biologically consistent pattern: there would appear to be an association regarding higher prevalence, severity, and progression rates of periodontitis among subjects with OSA.

The evidence obtained from this review emphasizes some very important implications for practice. First, given that there appears to be an unfavorable outcome for periodontal health among patients with OSA, it would appear that incorporating periodontal examination as a diagnostic and therapeutic strategy for sleep‐breathing disorders would be appropriate. Moreover, as evidenced within large research analyses conducted by Keller et al. (2013), and more recently within research conducted by Ytzhaik et al. (2023), and Stazić et al. (2022), there does appear to be an accumulation of periodontitis among patients with documented OSA. Moreover, as there exist considerable pathophysiological links, such as inflammation and systemic metabolic disturbances, there does exist justification within cross‐disciplinary practice and research for better collaboration and cooperation at the intersection of periodontists, sleep specialists, and dentists. Moreover, evidence documented within Verhelst et al. (2022) contributes toward an observation and finding that risk factors for OSA should be screened among patients with severe periodontitis, as there does appear to be tremendous proportions of at‐risk patients within that sector.

### 4.1 Limitations

Despite the strength of the available evidence, several limitations of the present systematic review and meta‐analysis should be acknowledged. A considerable degree of methodological heterogeneity was observed among the included studies, particularly regarding diagnostic criteria and assessment methods for both obstructive sleep apnea (OSA) and periodontitis. While several investigations used objective diagnostic approaches such as polysomnography and Apnea–Hypopnea Index (AHI) measurements, others relied on validated questionnaire‐based screening tools, potentially contributing to variability in diagnostic accuracy and increasing methodological heterogeneity across studies. Similarly, periodontal disease definitions and severity classifications were not entirely consistent among the included investigations, which may have affected the comparability of the reported outcomes.

Furthermore, the predominance of cross‐sectional studies limits the possibility of establishing temporal and causal relationships between OSA and periodontitis. Consequently, although a significant association between the two conditions was consistently observed, it remains unclear whether one condition may directly contribute to the onset or progression of the other. This limitation also restricts the ability to draw definitive conclusions regarding the long‐term effects of therapeutic or preventive interventions targeting either OSA or periodontal disease.

In addition, adjustment for important confounding variables—including age, body mass index (BMI), smoking status, diabetes mellitus, oral hygiene habits, and systemic comorbidities—varied substantially among studies. Residual confounding may therefore have influenced the strength of the observed associations. Finally, several studies included highly specific populations, such as obese individuals, patients recruited from dental or sleep clinics, or selected community cohorts, which may limit the generalizability of the findings to the broader population. Future prospective longitudinal studies using standardized diagnostic criteria and adequately controlled confounding factors are needed to better clarify the biological, immunological, and temporal relationship between OSA and periodontitis.

### 4.2 Future Directions

Despite these challenges, there should be focus on conducting longitudinal research that will be capable of addressing temporal and causality associations between OSA and periodontitis. Clinical trials that will evaluate the impact of OSA treatment, with a focus on CPAP therapy, on periodontitis outcomes, and perhaps additionally investigate the impact of periodontitis treatment on sleep breathing disorders, would be highly appreciated. A standardization of diagnostic criteria for these two conditions would make research highly uniform. Moreover, research on mechanistic paths, like inflammation and microbial imbalance and immunological interactions, would be highly appreciated and would be capable of understanding biologically these intricate connections.

## 5 Conclusions

From this meta‐analysis, it appears there is a clinically significant and biologically plausible link between periodontitis and OSA, with a risk of periodontitis nearly doubling for patients with OSA. Although it is not yet possible to confirm a causal relationship, consistency and overlap in mechanistic links, primarily mediated by inflammation and microorganisms, emphasize the importance of an interdisciplinary approach with periodontitis screening in patients with OSA and risk evaluation for OSA among patients with periodontitis.

## Author Contributions

Conceptualization: Francesca Sfondrini and Elena D'Angelo Methodology: Fariba Esperouz. Software: Giuseppina Raffaella Giannini Validation: Sara Ruffino and Michele Tepedino Formal analysis: Michele Tepedino Investigation: Mauro Lorusso Resources: Elena D'Angelo Data curation: Fariba Esperouz. Writing – original draft preparation: Fariba Esperouz and Giuseppe Burlon Writing – review and editing: Mauro Lorusso and Giuseppina Raffaella Giannini. Visualization: Lucio Lo Russo Supervision: Lucio Lo Russo and Domenico Ciavarella Project administration. Domenico Ciavarella: funding acquisition.

## Funding

The authors have nothing to report.

## Ethics Statement

The authors have nothing to report.

## Consent

The authors have nothing to report.

## Conflicts of Interest

The authors declare no conflicts of interest.

## Data Availability

The raw data supporting the conclusions of this article will be made available by the authors on request.
